# Oral Health Status of the Elderly Population in Iran

**DOI:** 10.1002/cre2.70170

**Published:** 2025-07-06

**Authors:** Shervan Shoaee, Mohammad‐Hossein Heydari, Kiarash Parchami, Leili Alizadeh, Soheila Darmiani, Shima Bijari, Parvin Parvaie, Farshad Sharifi, Ali Sharifi, Mohammad Reza Khami, Shayan Sobhaninejad

**Affiliations:** ^1^ Research Center for Caries Prevention, Dentistry Research Institute, School of Dentistry Tehran University of Medical Sciences Tehran Iran; ^2^ Elderly Health Research Center, Endocrinology and Metabolism Population Sciences Institute Tehran University of Medical Sciences Tehran Iran; ^3^ Non‐communicable Diseases Research Center, Endocrinology and Metabolism Research Institute Tehran University of Medical Sciences Tehran Iran; ^4^ Dental Research Center, Research Institute of Dental Sciences Shahid Beheshti University of Medical Sciences Tehran Iran; ^5^ Department of Oral and Maxillofacial Pathology, Faculty of Dentistry Birjand University of Medical Sciences Birjand Iran; ^6^ Department of Endodontics, Faculty of Dentistry Birjand University of Medical Sciences Birjand Iran; ^7^ Department of Oral and Maxillofacial Disease, School of Dentistry Birjand University of Medical Sciences Birjand Iran

**Keywords:** DMFT, edentulism, elderly, geriatrics, Iran, oral health, tooth loss

## Abstract

**Objectives:**

To evaluate the oral, periodontal, and dental health of the elderly population of Iran.

**Material and Methods:**

This was a cross‐sectional study as a part of the second wave of Birjand Longitudinal Aging Study (BLAS), which is a community‐based prospective cohort study. The comprehensive geriatric oral health assessment tool was used for data collection. Data was collected through clinical examinations.

**Results:**

Among the 1017 participants, the mean DMFT was 27.04. 67% (*n* = 681) were periodontally healthy, while mild to moderate periodontitis was diagnosed in 33% (*n* = 336) and severe periodontitis in 2.4% (*n* = 24). Xerostomia was diagnosed in 30% (305 individuals). 56.10% (*n* = 570) had removable dentures, among which 30.21% (*n* = 172) had poor retention, and 36.26% (*n* = 207) had poor stability. Red/white and exophytic lesions were diagnosed in 18.36% (*n* = 187) and 11.35% (*n* = 115), respectively. Tooth loss was prevalent, with 12.19% (*n* = 124) having mild tooth loss, 20.35% (*n* = 207) mild to moderate tooth loss, and 67.45% (*n* = 686) experiencing severe tooth loss.

**Conclusions:**

Our study revealed significant oral health challenges among the elderly population. High DMFT and the prevalence of xerostomia, periodontitis, tooth loss and poorly fitted dentures underscore the need for targeted dental care interventions. Comprehensive strategies, including raising awareness and improving access to oral healthcare, are essential to enhance the quality of life and overall health of the elderly population.

## 1 Introduction

The demographic shift of the elderly population is a global issue with its cultural, economic, and health‐related impacts (Abud et al. 2009). According to current trends, by 2050, the elderly population in developing countries will constitute 80% of the worldwide elderly demographic (Cheng et al. 2020; WHO 2012). In 2020, there were approximately 1 billion people aged 60 and over, a number that is projected to reach 2 billion by 2050 (Cheng et al. 2020; World Health Organization 2020). Among various age groups, the elderly are at a higher risk for chronic non‐communicable diseases such as oral‐related conditions, including periodontal diseases, tooth loss, edentulism, and dental caries (Barrio‐Cortes et al. 2021; Gil‐Montoya et al. 2015). From 1990 to 2015, the global prevalence and burden of untreated caries, severe periodontitis, and tooth loss significantly increased, with the elderly population being particularly affected by these changes (Kassebaum et al. 2017). According to the World Health Organization's 2022 country profile for Iran, the estimated prevalence of severe periodontal disease among individuals aged 15 years and older is 14.3%. In another provincial study involving individuals aged 35–70 years, it was observed that periodontal health indices worsened significantly with age. Bleeding on probing, periodontal pocket depth, and clinical attachment loss were all higher in older age groups. Additionally, poor oral hygiene and lower educational levels were associated with worse periodontal health (Baniasadi et al. 2021; Esfahanizadeh et al. 2013).

In Iran, national oral health surveys in 1990 and 2012 reported the mean Decayed, Missing, and Filled Teeth (DMFT) index for the elderly population to be 26.1 and 25.72, respectively, highlighting the poor dental health status among the Iranian elderly (Khoshnevisan et al. 2018; Shoaee et al. 2024a; Shoaee et al. 2024b). More recent estimations reported the DMFT index for the elderly Iranian population to be 26.84, with missing teeth (M) being 24.83 and comprising 92.5% of the total DMFT (Khoshnevisan et al. 2018; Shoaee et al. 2024a; Shoaee et al. 2024b). Studies have shown that elderly individuals with more than 20 teeth report higher levels of oral satisfaction along with better function and mastication (Khoshnevisan et al. 2018; Shoaee et al. 2024a; Shoaee et al. 2024b). Studies also indicate that demographic characteristics such as gender, age, education, and socioeconomic status can significantly impact oral health (Ekanayake and Perera 2004; Heydecke et al. 2004; Pallegedara and Ekanayake 2005; Steele et al. 2004).

The World Health Organization's Global Oral Health Program has prioritized improving oral health among the elderly since 2005. Along with this initiative, a recent 2021 commentary by WHO on global oral health promotion strategies emphasizes the importance of data‐driven and informed decision‐making and the importance of this step in achieving the goal of better global oral well‐being (Dye et al. 2022; Guarnizo‐Herreño et al. 2024; Petersen et al. 2013; Petersen and Yamamoto 2005; Watt and Aida 2022; Watt et al. 2020). Hence, in alignment with WHO's initiative and the current lack of comprehensive information and accurate tools to assess the oral health status of the elderly, this study aimed to evaluate the oral, periodontal, and dental health of this population in Birjand city using the Comprehensive Geriatric Oral Health Assessment Tool (CGOHAT). This novel tool was specifically designed for the evaluation of the oral health of the elderly. It comprises both clinical and self‐reported assessments.

## 2 Materials and Methods

This cross‐sectional study was a part of the second wave of the Birjand Longitudinal Aging Study (BLAS), which is a community‐based prospective cohort study (Heydari et al. 2024; Moodi et al. 2020). BLAS included 1420 elderly (age above 60) residents of Birjand county, selected through random cluster sampling among urban residents. Further information regarding the inclusion/exclusion criteria, sampling methods, and baseline data gathering procedures can be found in the BLAS's study protocol (Heydari et al. 2024; Moodi et al. 2020).

### 2.1 Study Design and Participants

As mentioned earlier, oral health assessment was part of the second wave of BLAS. We used the comprehensive geriatric oral health assessment tool (CGOHAT), which has been previously designed and validated (Shoaee et al. 2023). Further information regarding the CGOHAT and its domains is available elsewhere (Shoaee et al. 2023).

During the second wave, the study participants underwent various medical examinations in one of the affiliated hospitals of the Birjand Medical School. Those participants who were bedridden, had severe cognitive impairment, Alzheimer's disease, were unable to communicate, or had a life expectancy of less than 6 months were excluded from the study and were not included in the oral examination.

### 2.2 Data Collection and Oral Examination

The oral examinations were performed by four calibrated faculty members of the Birjand Medical School. The examination team included one oral and maxillofacial pathologist, two endodontics specialists, one oral medicine specialist, and a dental assistant. During examination, the dentist performed the clinical examination and the assistant entered the data in a pre‐designed online data gathering tool on a server at the Endocrinology and Metabolism Research Institute, Tehran University of Medical Sciences, Tehran, Iran. The recorded data was checked for validation on a daily basis.

### 2.3 Outcomes of Interest

The outcomes were defined based on the domains of the CGOHAT, covering (1) dental status and root caries; (2) periodontal status; (3) removable denture status; (4) oral mucosa and lesions; as well as (5) temporomandibular joint (TMJ) status.

Dental status and tooth loss were assessed using DMFT. Tooth loss was categorized as: (1) dentate or mild tooth loss – lost up to 10 teeth, (2) moderate tooth loss – lost 11 to 20 teeth, and (3) severe tooth loss – lost more than 20 teeth. Gingivitis was diagnosed if there was bleeding on probing in at least one tooth during the examination. The WHO periodontal probe was used for periodontal examination as specified by CGOHAT. Pocket depths from 0 to 3.5 mm were considered as periodontally healthy. Shallow pockets were defined as those between 3.5 and 5.5 mm, while pockets deeper than 5.5 mm were classified as deep pockets. Mild to moderate periodontitis was defined by the presence of at least one tooth with a shallow pocket without any deep pockets, whereas severe periodontitis was defined by the presence of at least one tooth with a deep pocket.

Participants' dentures were assessed for both hygiene and functionality. Functionality was evaluated by examining stability, retention, and denture tooth wear. Hygiene was assessed by checking for the presence of food debris, calculus, plaque, and pigmentation. The examination of the oral mucosa included assessments for xerostomia, red and white lesions, ulcers, and exophytic lesions, with a note on whether the exophytic lesion was caused by removable dentures. Xerostomia was diagnosed using two separate clinical tests: the presence of a lobulated tongue and the buccal mirror test. Mild to moderate xerostomia was defined as a positive result in only one of these tests, while severe xerostomia was defined as a positive results in both tests. TMJ signs were identified as deviation, clicking, crepitus, and a maximum mouth opening of less than 30 mm. TMJ symptoms were recorded if tenderness was observed during function.

Denture retention was tested by the clinicians when trying to dislodge the denture vertically (e.g., by pulling upward gently on the maxillary denture). Suction should be felt when trying to dislodge the denture with a tug — indicating acceptable seal. For stability, gentle side‐to‐side pressure was applied on the occlusal surfaces. A well‐stabilized denture should not rock or lift.

### 2.4 Ethical Consideration

Informed written consent was obtained from all elderly participants in this study. Data was collected with the participants' full consent and were anonymized. Participation in the study was free of charge, and participants could withdraw from the research at any stage.

### 2.5 Data Cleaning and Statistical Analysis

**Figure 1:**
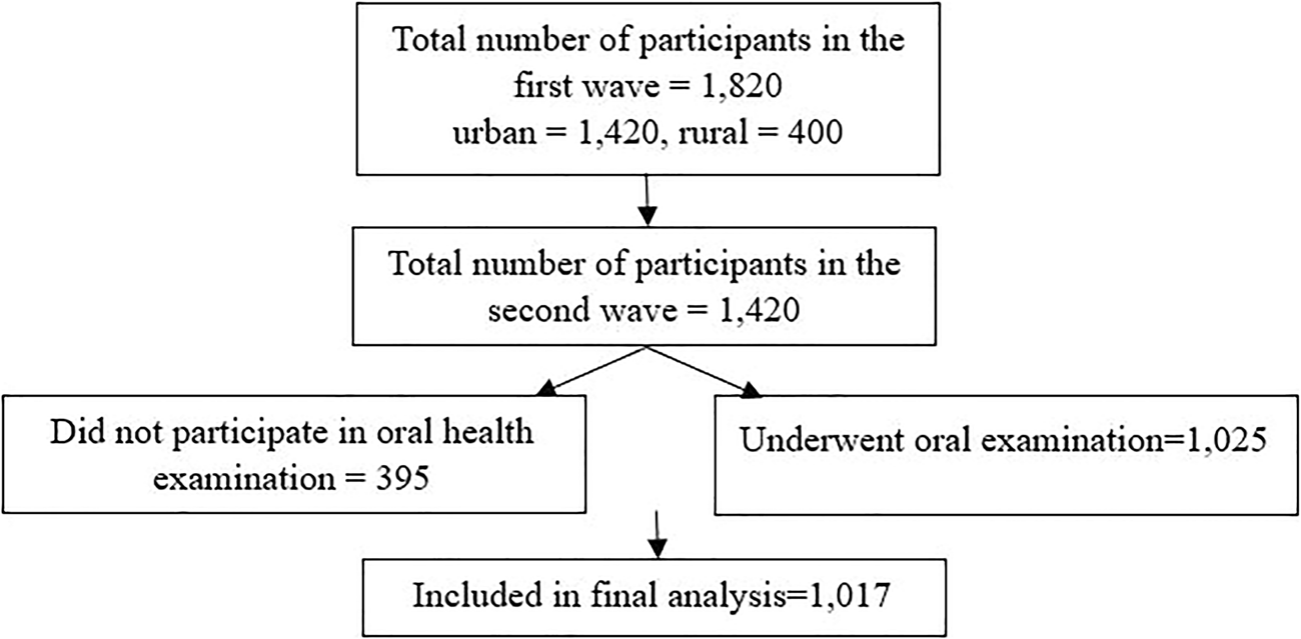
Inclusion of participants in the second wave of BLAS.

As mentioned before, data was recorded online using the DIGIT platform. In case of missing data, the examiners were contacted and if possible, the missing data was filled. If this was not possible, the record was excluded from the study. Among the 1025 participants that underwent oral examination based on the eligibility criteria (Figure 1), 1017 records were completed without missing data (missing rate less than 1%). Descriptive statistics (mean, standard deviation, *n*, and percentage) was used to report the outcomes. Chi‐square, *t*‐test, ANOVA, and MANOVA were used to compare the results among different groups. We used STATA V.17.0 (StataCorp LLC), R (R Foundation for Statistical Computing, Vienna, Austria), and RStudio (RStudio Inc., Boston, MA) for data cleaning, data analysis, and creating the figures.

## 3 Results

Overall, 1025 participants underwent clinical examination, among which 1017 records, comprising 510 females (50.15%) and 507 males (49.85%), were included in the final analysis. The mean age of participants was 72.42 years (Table 1).

**Table 1:** Oral disorders related outcomes.

		Female (*n* = 510)	Male (*n* = 507)	Both (*n* = 1,017)	*p* *
Age	Mean	71.65	73.20	72.42	**< 0.001**
	SD	6.83	6.16	6.54	
DMFT	Mean	27.09	26.98	27.04	*0.818*
	SD	7.38	8.10	7.75	
D	Mean	2.25	2.33	2.29	0.744
	SD	3.66	3.81	3.73	
	% of total DMFT	9.59%	10.86%	10.22%	
M	Mean	23.77	23.87	23.82	0.863
	SD	9.63	10.23	9.93	
	% of total DMFT	85.19%	85.04%	85.11%	
F	Mean	1.07	0.77	0.92	0.075
	SD	2.81	2.46	2.64	
	% of total DMFT	5.21%	4.09%	4.65%	
Tooth loss	Mild (1–10)	56 (1.98%)	68 (13.4%)	124 (12.19%)	0.232
	Moderate (11–20)	113 (22.16%)	94 (18.54%)	207 (20.35%)	
	Severe (21–32)	341 (66.86%)	345 (68.05%)	686 (67.45%)	
Gingivitis^a^	No	153 (58.40%)	131 (54.81%)	284 (56.69%)	0.419
	Yes	109 (41.60%)	108 (45.19%)	217 (43.31%)	
Periodontitis^a^	No	184 (70.23%)	159 (66.53%)	343 (68.46%)	0.122
	Mild to Moderate	69 (26.34%)	77 (32.22%)	146 (29.14%)	
	Severe	9 (3.44%)	3 (1.26%)	12 (2.40%)	
Dental calculus^a^	No	100 (38.17%)	103 (43.46%)	203 (40.68%)	0.229
	Yes	162 (61.83%)	134 (56.54%)	296 (59.32%)	
Gingival recession^a^	No	46 (17.62%)	42 (17.72%)	88 (17.67%)	0.977
	Yes	215 (82.38%)	195 (82.28%)	410 (82.33%)	
TMJ symptoms	No	419 (84.14%)	419 (84.99%)	838 (84.56%)	0.710
	Yes	79 (15.86%)	74 (15.01%)	153 (15.44%)	
TMJ signs	No	473 (94.98%)	478 (96.96%)	951 (95.96%)	0.114
	Yes	25 (5.02%)	15 (3.04%)	40 (4.04%)	
Lobulated tongue	No	477 (96.36%)	465 (94.70%)	942 (95.54%)	0.207
	Yes	18 (3.64%)	26 (5.30%)	44 (4.46%)	
Mirror test	No	448 (90.51%)	455 (92.29%)	903 (91.40%)	0.317
	Yes	47 (9.49%)	38 (7.71%)	85 (8.60%)	
Xerostomia	No	442 (89.47%)	442 (89.84%)	884 (89.66%)	0.926
	Mild to Moderate	39 (7.89%)	36 (7.32%)	75 (7.61%)	
	Severe	13 (2.63%)	14 (2.85%)	27 (2.74%)	
Red/white lesions	No	414 (83.64%)	391 (76.63%)	805 (81.64%)	0.105
	Yes	81 (16.36%)	100 (20.37%)	181 (18.36%)	
Exophytic lesions	No	444 (89.70%)	431 (87.60%)	875 (88.65%)	0.299
	Yes	51 (10.30%)	61 (12.40%)	112 (11.35%)	
Oral ulcer	No	466 (93.95%)	462 (93.90%)	928 (93.93%)	0.974
	Yes	30 (6.05%)	30 (6.10%)	60 (6.07%)	

Denture features		Female (*n* = 270)	Male (*n* = 251)	Both (*n* = 521)	*p*
Color pigment	No	113 (41.39%)	93 (36.90%)	206 (39.24%)	0.293
	Yes	160 (58.61%)	159 (63.10%)	319 (60.76%)	
Plaque	No	143 (52.38%)	125 (50%)	268 (51.24%)	0.586
	Yes	130 (47.62%)	125 (50%)	255 (48.76%)	
Calculus	No	196 (71.79%)	164 (65.34%)	360 (68.70%)	0.111
	Yes	77 (28.21%)	87 (34.66%)	164 (31.30%)	
Food retention	No	206 (75.46%)	165 (65.74%)	371 (70.80%)	**0.014**
	Yes	67 (24.54%)	86 (34.26%)	153 (29.20%)	
Retention	No	190 (69.60%)	175 (70.00%)	365 (69.79%)	0.920
	Yes	83 (30.40%)	75 (30%)	158 (30.21%)	
Stability	No	178 (65.20%)	156 (62.15%)	334 (63.74%)	0.468
	Yes	95 (34.80%)	95 (37.85%)	190 (36.26%)	
Tooth abrasion	No	192 (71.11%)	179 (71.31%)	371 (71.21%)	0.959
	Yes	78 (28.89%)	72 (28.69%)	150 (28.79%)	

		DMFT			*p*
		Mean	SD		
Age	60–64	25.53	±8.00		**< 0.001**
	65–69	24.43	±8.81		
	70–74	27.06	±7.84		
	75–79	29.28	±5.83		
	80–84	30.51	±4.11		
	85 or Older	31.23	±2.64		

		Periodontitis			*p*
		No, *n* (%)	Mild to Moderate, *n* (%)	Severe, *n* (%)	
Age	60–64	32 (9.33%)	16 (10.96%)	0 (0%)	0.060
	65–69	134 (39.07%)	68 (46.86%)	7 (58.33%)	
	70–74	98 (28.57%)	31 (21.23%)	2 (16.67%)	
	75–79	52 (15.16%)	20 (13.70%)	0 (0%)	
	80–84	19 (5.54%)	8 (5.48%)	1 (8.33%)	
	85 or Older	8 (2.33%)	3 (2.05%)	2 (16.67%)	

		Tooth Loss			*p*
		Mild, *n* (%)	Mild, *n* (%)	Mild, *n* (%)	
Age	60–64	12 (15.19%)	25 (31.65%)	42 (53.16%)	**< 0.01**
	65–69	71 (21.52%)	86 (26.06%)	173 (52.42%)	
	70–74	28 (10.04%)	58 (20.79%)	193 (69.18%)	
	75–79	9 (5.17%)	23 (13.22%)	142 (81.61%)	
	80–84	3 (3.33%)	10 (11.11%)	77 (85.56%)	
	85 or Older	1 (1.54%)	5 (7.69%)	59 (90.77%)	
Have denture	No	93 (94.90%)	171 (82.61%)	171 (24.93%)	**< 0.01**
	Yes	5 (5.10%)	36 (17.39%)	515 (75.07%)	

*Note:* Bold *p* values indicate statistical significance.

^a^ *n* = 541 (those with *M* = 32, were excluded).

* *p* for *t*‐test and chi^2^ test.

**Figure 2:**
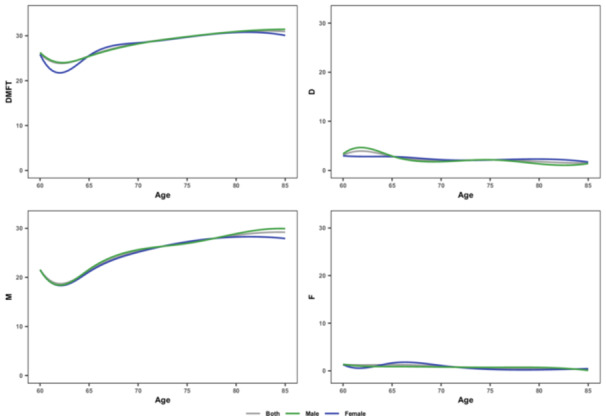
Trend of DMFT and its components among different age groups.

**Figure 3:**
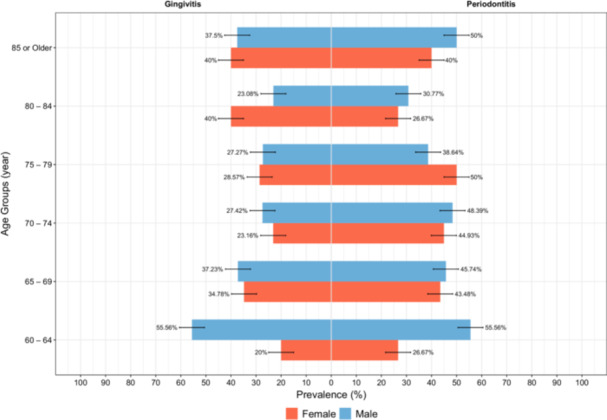
Pyramid chart of the prevalence of periodontal conditions among different age groups.

The mean DMFT was 27.04 (± 6.54). The mean DMFT was 27.09 (± 7.38) for females and 26.98 (± 8.10) for males(*p* = *0.818*). More than 85% of the DMFT was comprised of Missing teeth in both genders. The mean M was 23.77 (± 9.63) for females and 23.87 (± 10.23) for males, with no significant difference (*p* = *0.863*). The mean DMFT showed and increasing trend from 60 to 64 years to 85 years or older age groups, except for the 65 to 69 years old group (Table 1, Figure 2). Mild to moderate periodontitis was less prevalent in females (26.34%) compared to males (32.22%), and severe periodontitis was more prevalent in females (3.44%) than in males (1.26%) with no significant gender differences (*p*‐value = 0.122). Figure 3 shows the prevalence of periodontitis among different age groups.

**Figure 4:**
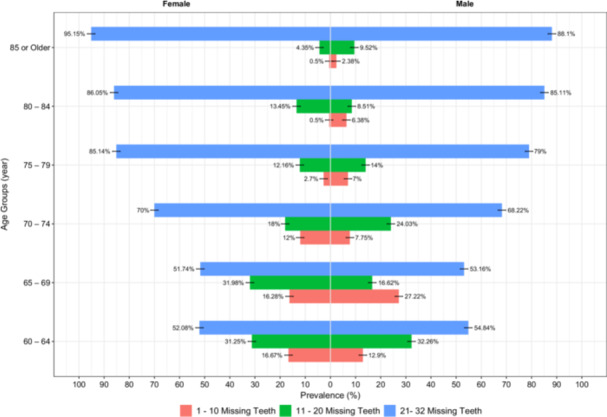
Pyramid chart of the prevalence of tooth loss among different age groups.

Regarding tooth loss (Table 2, and Figure 3), 56 females (1.98%) and 68 males (13.4%) had mild tooth loss, 113 females (22.16%) and 94 males (18.54%) had moderate tooth loss, and 341 females (66.86%) and 345 males (68.05%) had severe tooth loss (*p* = *0.232*). Also, tooth loss showed a similar pattern as DMFT, and the prevalence of moderate and severe tooth loss increased with age (Figure 4), with 90.77% of the participants in 85‐or‐older age group having severe tooth loss (*p* < *0.001*). Among those with moderate or severe tooth loss (Appendices 1 and 2), 171 (82.61%) and 171 (24.93%) did not have removable denture, respectively (*p* < *0.001*). Among those who had denture, 158 (30.21%) dentures had poor retention and 190 (36.26%) had poor stability (Tables 2, 3, Appendix 3). Also, 15.44% reported TMJ symptoms, with 15.86% of females and 15.01% of males affected, showing no significant gender difference (*p* = *0.710*). Clinical TMJ signs were observed in 4.04% of participants, including 5.02% of females and 3.04% of males, also with no significant difference between genders (*p* = *0.114*). Further information regarding oral lesions and xerostomia is summarized in Table 1 and Appendix 4.

**Table 2:** DMFT, periodontitis, tooth loss, and denture use among different age groups population.

		DMFT				*p*‐value
		Mean		SD		
Age	60–64	25.53		±8.00		**< 0.001**
	65–69	24.43		±8.81		
	70–74	27.06		±7.84		
	75–79	29.28		±5.83		
	80–84	30.51		±4.11		
	85 or Older	31.23		±2.64		

		Periodontitis				
		No, *n* (%)	Mild to Moderate, *n* (%)		Severe, *n* (%)	
Age	60–64	32 (9.33%)	16 (10.96%)		0 (0%)	0.060
	65–69	134 (39.07%)	68 (46.86%)		7 (58.33%)	
	70–74	98 (28.57%)	31 (21.23%)		2 (16.67%)	
	75–79	52 (15.16%)	20 (13.70%)		0 (0%)	
	80–84	19 (5.54%)	8 (5.48%)		1 (8.33%)	
	85 or Older	8 (2.33%)	3 (2.05%)		2 (16.67%)	

		Tooth Loss				
		Mild, *n* (%)	Moderate, *n* (%)		Severe, *n* (%)	
Age	60–64	12 (15.19%)	25 (31.65%)		42 (53.16%)	**< 0.001**
	65–69	71 (21.52%)	86 (26.06%)		173 (52.42%)	
	70–74	28 (10.04%)	58 (20.79%)		193 (69.18%)	
	75–79	9 (5.17%)	23 (13.22%)		142 (81.61%)	
	80–84	3 (3.33%)	10 (11.11%)		77 (85.56%)	
	85 or Older	1 (1.54%)	5 (7.69%)		59 (90.77%)
Have denture	No	93 (94.90%)	171 (82.61%)		171 (24.93%)	**< 0.001**
	Yes	5 (5.10%)	36 (17.39%)		515 (75.07%)	

*Note:* Bold *p* values indicate statistical significance.

**Table 3:** Denture status among the study population.

Denture's feature^a^		Female (*n* = 270)	Male (*n* = 251)	Both (*n* = 521)	*p*‐value^b^
Color pigment	No	113 (41.39%)	93 (36.90%)	206 (39.24%)	0.293
	Yes	160 (58.61%)	159 (63.10%)	319 (60.76%)	
Plaque	No	143 (52.38%)	125 (50%)	268 (51.24%)	0.586
	Yes	130 (47.62%)	125 (50%)	255 (48.76%)	
Calculus	No	196 (71.79%)	164 (65.34%)	360 (68.70%)	0.111
	Yes	77 (28.21%)	87 (34.66%)	164 (31.30%)	
Food retention	No	206 (75.46%)	165 (65.74%)	371 (70.80%)	**0.014**
	Yes	67 (24.54%)	86 (34.26%)	153 (29.20%)	
Retention	No	190 (69.60%)	175 (70.00%)	365 (69.79%)	0.920
	Yes	83 (30.40%)	75 (30%)	158 (30.21%)	
Stability	No	178 (65.20%)	156 (62.15%)	334 (63.74%)	0.468
	Yes	95 (34.80%)	95 (37.85%)	190 (36.26%)	
Tooth abrasion	No	192 (71.11%)	179 (71.31%)	371 (71.21%)	0.959
	Yes	78 (28.89%)	72 (28.69%)	150 (28.79%)	

*Note:* Bold *p* value indicates statistical significance.

^a^ Those without denture were excluded.

^b^ *p* for chi^2^ test.

## 4 Discussion

Our study aimed to evaluate the oral health status of the elderly population in Birjand, Iran, using the Comprehensive Geriatric Oral Health Assessment Tool (CGOHAT). Assessing the oral health of the elderly is crucial for preventing severe oral diseases and associated systemic issues. Consequently, it is imperative for all components of the healthcare system responsible for maintaining public oral health to be cognizant of the specific needs of the elderly population. In other words, oral health of the elderly population can represent the end result of oral health policy planning in a healthcare system (Chávez et al. 2022; Miyazaki et al. 2017).

It is important to note that several assessment tools have been developed to evaluate the oral health status of the elderly. In this study, we utilized the CGOHAT for oral health assessment as it assists clinicians in performing a more comprehensive evaluation of their elderly patients' oral health, compared to other available tools (Shoaee et al. 2023). To conclude the findings of our study, we can mention that the majority of our study population (54%) were edentulous and lost their dentition due to caries or periodontal disease. Even among the dentate population, the majority (80%) either suffered from severe tooth loss (lost at least 21 teeth). Among those with severe tooth loss, more than 20% did not have a denture, and among those with dentures, at least one‐third suffered from poor retention and stability. More importantly, the mean DMFT was 27, with mean D, M, and F being 2, 24, and 1. Around 10% suffered from xerostomia, and more than one‐fourth of the population had an exophytic lesion, a red/white lesion, or a ulcer in their mouth.

Our results revealed a significant disparity in the prevalence of gingivitis, mild to moderate periodontitis, and severe periodontitis. Approximately 43% of participants were found to have gingivitis, 30% had mild to moderate periodontitis, and 2.5% had severe periodontitis. The high prevalence of gingivitis and mild periodontitis highlights the persistent presence of inflammation, which likely contributed to high grades of tooth mobility, which in turn, may have necessitated tooth extractions at younger ages, as indicated by the high mean of the M component in our study population. Thus, the low prevalence of severe periodontitis among our study population cannot be attributed to good oral health or healthy periodontium, however, it actually shows how early the periodontal inflammation starts and how early it leads to extraction and loss of functional units, that by the time they are 60 years old, half of the elderly population lost at least 20 teeth and by 85 years of age, more than 80% of them (Figure 4).

Severe tooth loss remains a significant national health concern, as evidenced by our study, that over two‐thirds of participants were affected. This trend is consistent with reports from various regions. For example, studies have indicated that nearly 70% of the elderly population in Spain experience severe tooth loss, also in Saudi Arabia and Lebanon, prevalence rates among the elderly are approximately 60% and 65%, respectively (Al‐Ansari 2014; Andari et al. 2022; Hakeem et al. 2024; Morales‐Suárez‐Varela et al. 2011). These studies highlight the widespread prevalence of edentulism among older adults across both developed and developing countries.

As mentioned earlier, one‐fourth of our study population with severe tooth loss do not have dentures. Also, as it is evident in Appendices 1 and 2, tooth loss increases as age increases, and more importantly, the use of dentures decreases. This highlights the fact that as people get older, loose more teeth, and require more immediate care to restore their masticatory function. However, it is less likely for them to receive proper treatment. Also, prior dissatisfaction due to a denture with suboptimal functionality may also be a contributing factor for not having or using dentures. Similar patterns have been seen across various studies. 25% of elderly individuals with significant tooth loss similarly do not use dentures (Heydecke et al. 2004; Pallegedara and Ekanayake 2005; Yen et al. 2015). Denture use patterns show some variation but highlight common global challenges. For instance, in China, 54% of the elderly population wear dentures, and also 33% of denture users in Japan reported a negative impact on their oral health‐related quality of life (OHRQoL) (Techapiroontong et al. 2022; Yen et al. 2015). In a study in Iran, 40% of elderly individuals had dentures; however, many faced functional difficulties, with 30.21% experiencing retention issues and 36.26% stability issues (Jamalinasab et al. 2024; Omidpanah et al. 2021). Additionally, 22.8% of denture users in Iran suffer from oral lesions, often linked to poor hygiene practices or ill‐fitting dentures (Jamalinasab et al. 2024; Omidpanah et al. 2021). Similar trends were observed in Portugal, where 21% of elderly denture users reported comparable complications (Desai and Nair 2023; Prakash et al. 2022).

Based on our results, the mean DMFT index was 27.04, highlighting a significant oral disease burden in this group. Notably, the M component accounted for over 85.11% of the total DMFT index in both males and females, indicating that tooth loss is the predominant dental condition among the elderly population and the major determinant of poor oral health (Shoaee et al. 2024a). Other national studies reported similar estimated patterns, with one meta‐analysis reporting the mean DMFT to be 25.72 for elderly individuals (Shoaee et al. 2024a). Another study estimated the mean DMFT to be 24.9 among people who were 60 years or older (Shoaee et al. 2024b). Similar to our study, the M component comprises more than 90% of the total DMFT index in these studies.

When comparing our results to other regional and global studies, notable variations in DMFT values become apparent. For instance, a study from Turkey reported a mean DMFT of 19.1, while another study of elderly residents in a Turkish homecare indicated a significantly higher DMFT of 29.3 (Bozdemir et al. 2016; Ünlüer et al. 2007). These comparisons highlight substantial regional variability and emphasize the considerable burden of oral disease among the elderly population in Middle Eastern countries. In contrast, developed regions such as Hong Kong have reported lower DMFT values, with a mean of 17.6, likely reflecting improved oral health outcomes due to greater access to dental care and preventive interventions (Chan et al. 2021; Chu et al. 2015; Lo and Schwarz 1994; Yang et al. 2021). In Saudi Arabia, DMFT values ranged between 18.6 and 24.3 (Al‐Ansari 2014; Alshammari et al. 2021), whereas in Lebanon, the mean DMFT was 23.1 (Andari et al. 2022; Choufani et al. 2020). This finding aligns with a similar study reporting a mean DMFT index of approximately 26, with missing teeth comprising more than 80% of the total score in the elderly population in Myanmar (Knodel and Teerawichitchainan 2017; Zin et al. 2020).

The implications of inadequate oral hygiene and tooth loss significantly impact the overall quality of life and systemic health among the elderly (Azami‐Aghdash et al. 2021; Pakize et al. 2020; Paredes‐Rodríguez et al. 2016). Inadequate oral health is frequently linked to nutritional deficiencies, unsatisfactory masticatory function, diminished quality of life, and an elevated risk of systemic diseases, such as cardiovascular disease and diabetes, which are prevalent among older adults (Chan et al. 2024; Di Spirito 2022; Poudel et al. 2024). Despite the considerable burden of oral diseases, barriers including insufficient awareness and restricted access to dental care persist as major challenges in addressing these concerns in Iran. Addressing these oral health concerns necessitates a multifaceted approach, which includes increasing awareness, enhancing dental care services, implementing designated policies to address such shortcomings, and incorporating oral health into the broader healthcare system for the elderly. Global comparisons further emphasize the variability in oral health outcomes among older adults (Chávez et al. 2022), underscoring the necessity for targeted interventions and customized healthcare policies to enhance oral health and quality of life for this population.

As mentioned earlier, the WHO ignited its initiative for improving the oral health of the elderly around two decades age, however, the oral health policies in Iran and other countries, still have not developed targeted policies for this group. Our oral health policies still focus on children younger than 12 years old and pregnant women as their primary target groups. This study and similar recent literature strongly suggest that these policies should be revisited and revised accordingly (Bayat 2018; Mohammadpour et al. 2020; Pakshir 2004).

Our study offers valuable insights into the oral health status of the elderly in Iran through its well designed and structured comprehensive data collection. Its strengths include addressing a critical research question about elderly oral health, utilizing a validated and comprehensive assessment tool (CGOHAT), and involving trained and calibrated examiners, which enhances the reliability of the findings. However, the study also has limitations. It focused exclusively on urban residents due to COVID‐19‐related constraints, potentially limiting the generalizability to rural populations. The exclusion of bedridden individuals and those with severe cognitive impairment may introduce selection bias. While gender differences in oral health outcomes were observed, they were not statistically significant, and the high prevalence of tooth loss highlights the need for further investigation into barriers to dental care. It should be noted, as more than two thirds of our study population had severe tooth loss and more than half of them were edentulous, our data regarding periodontitis, gingivitis, dental calculus, and F component of the DMFT index were faced with certain limitations as they could only be calculated among the dentate population. These factors may have impacted our results and should be considered when interpreting the study's findings and their implications for public health interventions.

## 5 Conclusions

This study highlights the significant oral health challenges faced by the elderly population in urban areas of Iran. The findings underscore the high prevalence of dental caries, periodontitis, xerostomia, and denture‐related issues such as retention, stability, and oral lesions. These conditions not only affect the quality of life but also pose risks for systemic health problems. There is a clear necessity for oral health improvement strategies for the elderly population that include raising awareness, improving access to oral healthcare, and integrating oral health services into the broader general healthcare system for the elderly. Addressing these issues requires a multifaceted approach and further research to develop tailored healthcare policies. Such efforts are essential to improve the overall health and well‐being of the elderly population, both in Iran and globally.

## Author Contributions

**Shervan Shoaee:** design, conceptualization, data gathering, original draft, revision, interpreting the results, supervision. **Mohammad‐Hossein Heydari:** design, conceptualization, data curation and cleaning, data analysis, creating figures, interpreting the results, original draft, revision, supervision. **Kiarash Parchami:** interpreting the results, original draft, revision. **Leili Alizadeh:** data gathering, interpreting the results, revision. **Soheila Darmiani:** data gathering, interpreting the results, revision. **Shima Bijari:** data gathering, interpreting the results, revision. **Parvin Parvaie:** data gathering, interpreting the results, revision. **Farshad Sharifi:** data gathering, interpreting the results, revision, supervision. **Ali Sharifi:** data gathering, data cleaning, interpreting the results, revision. **Mohammad Reza Khami:** interpreting the results, revision, supervision. **Shayan Sobhaninejad:** data gathering, interpreting the results, original draft, revision, supervision.

## Ethics Statement

This study was approved by the ethics committee of Tehran University of Medical Sciences (IR.TUMS.DENTISTRY.REC.1398.181).

## Consent

Written consent was obtained from all participants before any data collection or oral examinations.

## Conflicts of Interest

The authors declare no conflicts of interest.

## Supporting information


**Appendix 1.** Age pattern of denture use based on severity of tooth loss.

**Appendix 2.** Pyramid chart showing the pattern of denture use among different age groups based on severity of tooth loss.

**Appendix 3.** Prevalence of oral lesions and xerostomia among different age groups.

**Appendix 4.** Prevalence of denture‐related problems among different age groups.

**Data.** Prevalence of denture‐related problems among different age groups.

## Data Availability

The data that supports the findings of this study are publicly available in the supporting material of this article.
