# Supplementary figures and images for: Oral Health Status of the Elderly Population in Iran
Source: Clin Exp Dent Res. 2025 Jul 6;11(4):e70170. doi: 10.1002/cre2.70170 (PMC12229249; doi:10.1002/cre2.70170)

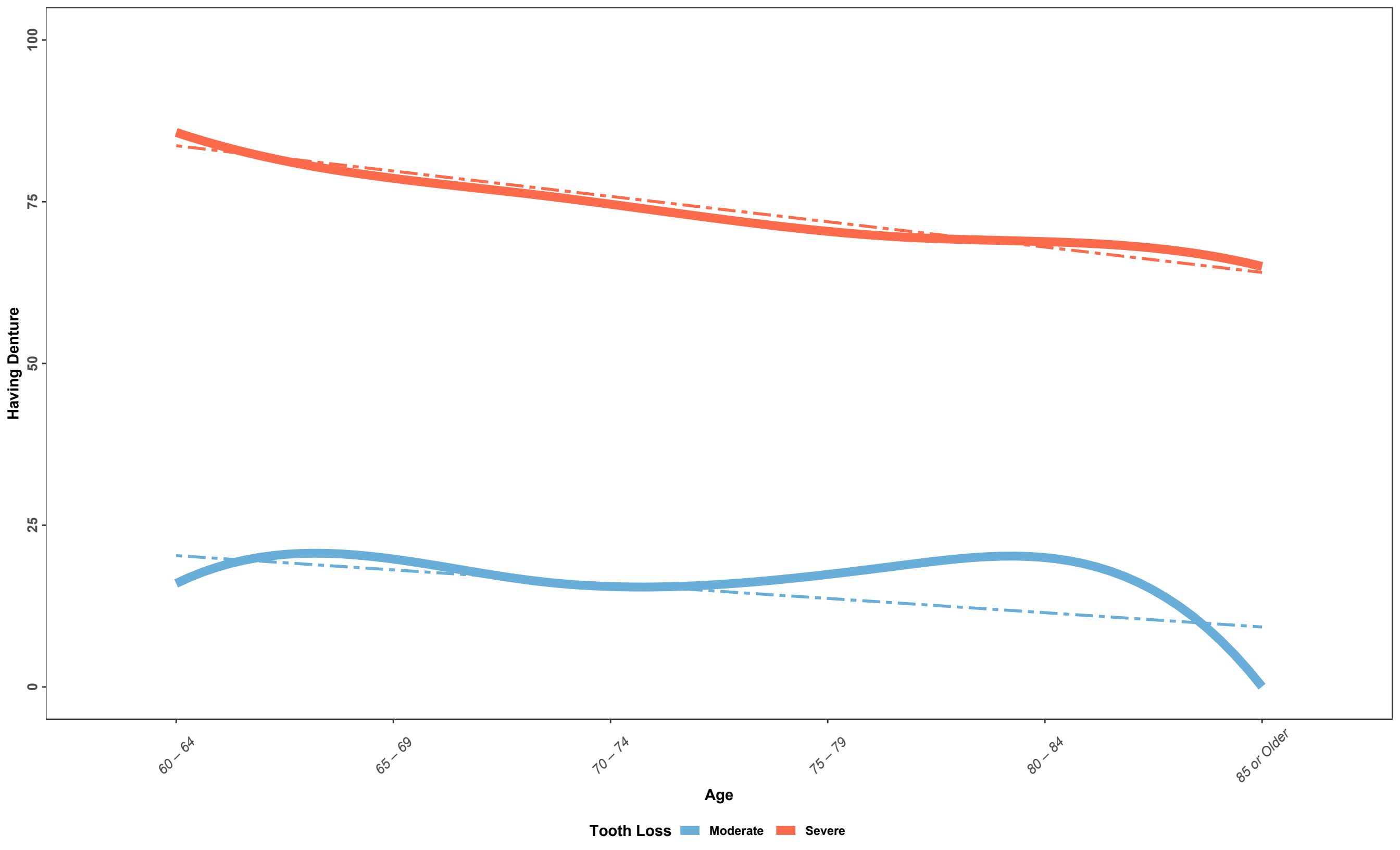

Supplement: Supplementary file 1 — Appendix 1. Age pattern of denture use based on severity of tooth loss. [file CRE2-11-e70170-s005.pdf]

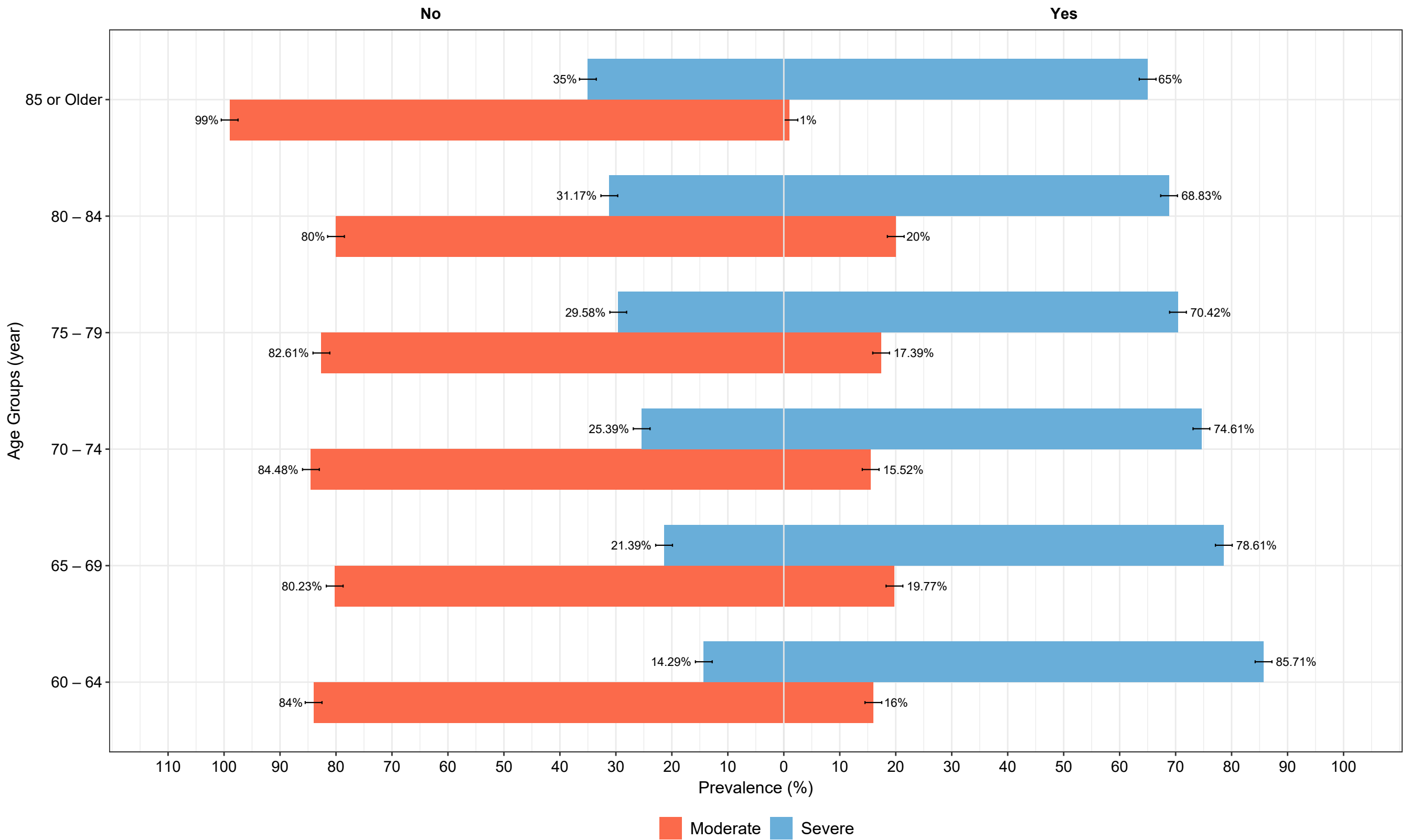

Supplement: Supplementary file 2 — Appendix 2. Pyramid chart showing the pattern of denture use among different age groups based on severity of tooth loss. [file CRE2-11-e70170-s001.pdf]

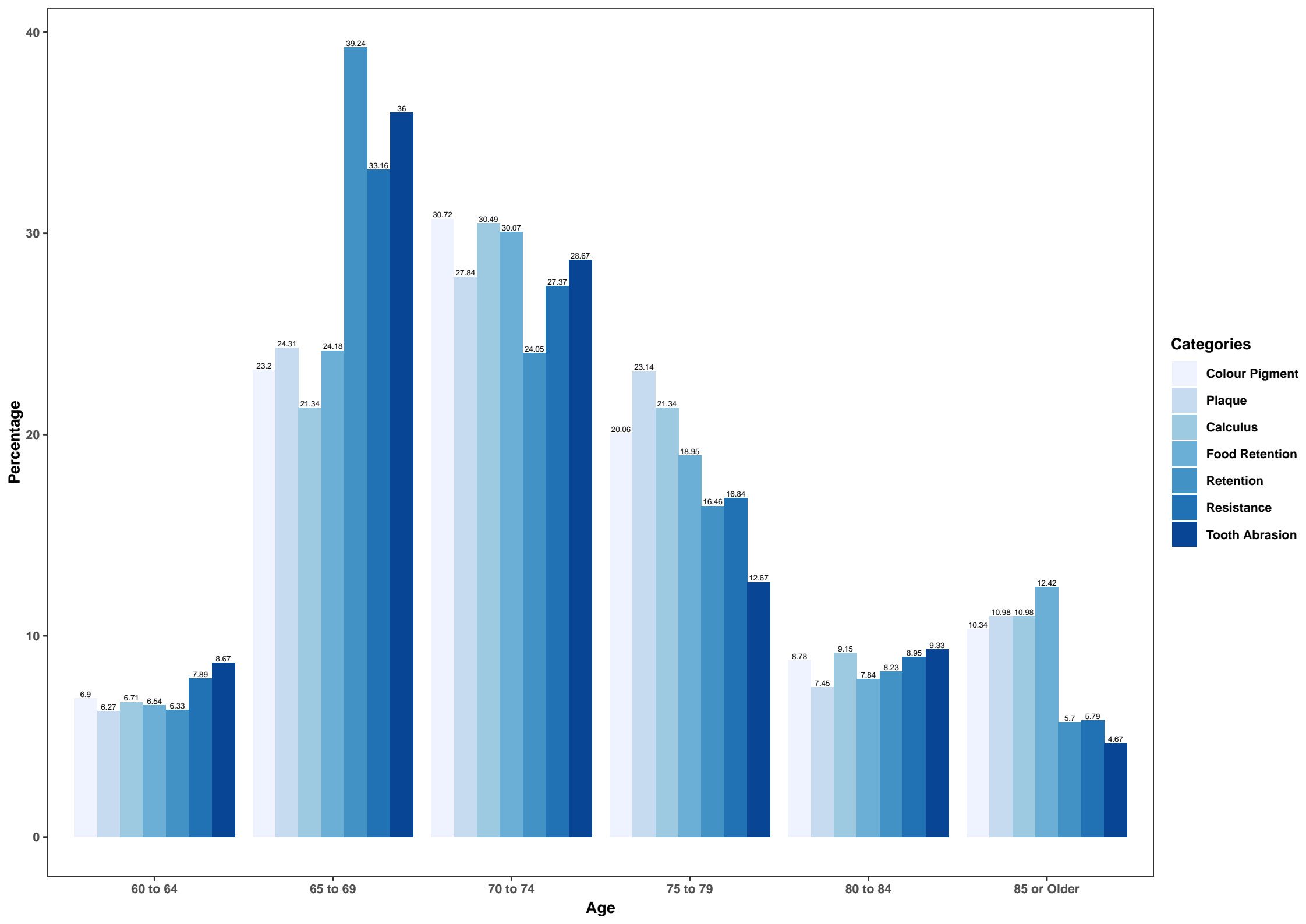

Supplement: Supplementary file 3 — Appendix 3. Prevalence of oral lesions and xerostomia among different age groups. [file CRE2-11-e70170-s002.pdf]

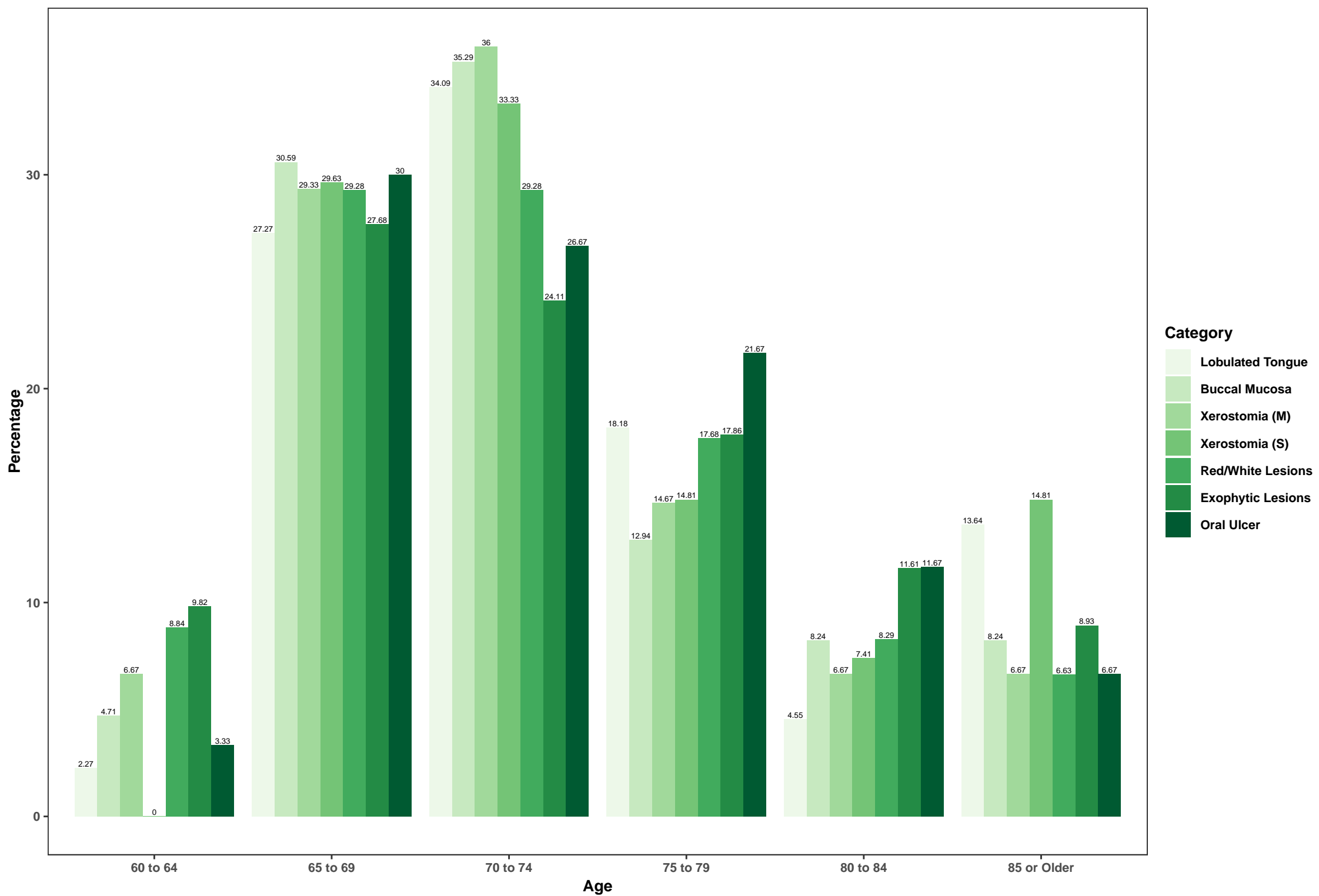

\*Xerostomia (M): Mild to Moderate; Xerostomia (S): Severe

Supplement: Supplementary file 4 — Appendix 4. Prevalence of denture‐related problems among different age groups. [file CRE2-11-e70170-s004.pdf]
